# Downregulation of m6A Methyltransferase in the Hippocampus of *Tyrobp*^–/–^ Mice and Implications for Learning and Memory Deficits

**DOI:** 10.3389/fnins.2022.739201

**Published:** 2022-03-21

**Authors:** Zhanyun Lv, Tongxiao Xu, Ran Li, Dejie Zheng, Yanxin Li, Wei Li, Yan Yang, Yanlei Hao

**Affiliations:** ^1^Zhejiang University Medical Center, Hangzhou, China; ^2^School of Brain Science and Brain Medicine, Zhejiang University, Hangzhou, China; ^3^College of Clinical Medicine, Jining Medical University, Jining, China; ^4^Cheeloo College of Medicine, Shandong University, Jinan, China; ^5^Health Management Center, Weifang People’s Hospital, Weifang, China; ^6^Department of Neurology, Pingdu People’s Hospital, Qingdao, China; ^7^Department of Neurology, The Affiliated Hospital of Jining Medical University, Jining, China

**Keywords:** *Tyrobp*^–/–^ mice, m6A methylation, METTL3, METTL14, WTAP, ALKBH5, FTO, MeRIP-seq

## Abstract

Loss-of-function mutations in the gene that encodes TYRO protein kinase-binding protein (*TYROBP*) cause Nasu-Hakola disease, a heritable disease resembling Alzheimer’s disease (AD). Methylation of N6 methyl-adenosine (m6A) in mRNA plays essential roles in learning and memory. Aberrant m6A methylation has been detected in AD patients and animal models. In the present study, *Tyrobp^–/–^* mice showed learning and memory deficits in the Morris water maze, which worsened with age. *Tyrobp^–/–^* mice also showed elevated levels of total tau, Ser202/Thr205-phosphorylated tau and amyloid β in the hippocampus and cerebrocortex, which worsened with aging. The m6A methyltransferase components METTL3, METTL14, and WTAP were downregulated in *Tyrobp^–/–^* mice, while expression of demethylases that remove the m6A modification (e.g., FTO and ALKBH5) were unaltered. Methylated RNA immunoprecipitation sequencing identified 498 m6A peaks that were upregulated in *Tyrobp^–/–^* mice, and 312 m6A peaks that were downregulated. Bioinformatic analysis suggested that most of these m6A peaks occur in sequences near stop codons and 3′-untranslated regions. These findings suggest an association between m6A RNA methylation and pathological TYROBP deficiency.

## Introduction

In the brain, TYRO protein kinase-binding protein (TYROBP) is expressed mainly by microglia (Ma et al., 2015). A *TYROBP*-centered pathway has been identified in microglia of healthy adult and aged mice, and 44 of the 100 genes interact directly or indirectly with *TYROBP* (Hickman et al., 2013). Loss-of-function mutations in the *TYROBP* gene cause Nasu-Hakola disease (NHD), also known as polycystic lipomembranous osteodysplasia with sclerosing leukoencephalopathy (MIM 221770) (Paloneva et al., 2000). NHD patients typically experience frequent bone fractures starting in their 30s, progressive memory deficits and personality changes starting from their 40s, and eventually dementia and death (Xing et al., 2015). *TYROBP* mutations have also been detected in patients with Alzheimer’s disease (AD) (Pottier et al., 2016). A study of 1,647 AD patients *post mortem* revealed upregulation of TYROBP and suggested that it is a key regulator of AD-related processes (Zhang et al., 2013).

NHD and AD overlap significantly in clinical presentations as well as in pathological features. The pathologic hallmark of AD is the accumulation of insoluble neurotoxic aggregates, including amyloid β (Aβ) plaques and intracellular tau neurofibrillary tangles. Aβ deposition and neurofibrillary changes have been reported in the brains of NHD patients bearing homozygous Q33X mutations in the *TREM2* gene (Maderna et al., 2021). NHD patients have been found to contain Aβ deposits in the frontal cortex and phosphorylated tau in hippocampal neurons (Satoh et al., 2018).

N6-methyladenosine (m6A) methylation in RNA is a post-transcriptional modification that attaches a methyl group at the N6 position of adenosine (Zaccara et al., 2019), which helps regulate the localization, transport and translation of mRNAs involved in memory and learning (Zhang et al., 2018). Transgenic *APP/PS1* mice show elevated m6A methylation in the cerebrocortex and hippocampus; upregulation of methyltransferase-like protein 3 (METTL3), which helps generate m6A (Liu et al., 2014); as well as downregulation of fat mass- and obesity-associated protein (FTO), a demethylase that removes the m6A modification (Jia et al., 2011; Han et al., 2020).

Given the similarities between AD and NHD, we compared hippocampal m6A methylation between *Tyrobp*^–/–^ and wild-type (WT) mice. The function of differentially expressed RNAs was predicted based on enrichment in Gene Ontology (GO) terms and Kyoto Encyclopedia of Genes and Genomes (KEGG) pathways.

## Materials and Methods

### Animal Subjects

F0 *Tyrobp*^+/–^ mice were obtained from Cyagen Biosciences (Guangzhou, China). The mouse strain was constructed by microinjecting into fertilized eggs a transcription activator-like effector nuclease (TALEN) that removes 10 bases (GTACAGGCCC) from exon 2 of the *TYROBP* gene. F0 mice were bred with C57/BL6 mice to produce the F1 generation, and the mutant F1 generation was inbred to generate the F2 generation. Gene knockout was confirmed using Sanger sequencing and western blotting.

All experiments were carried out using male mice. Age-matched WT littermates were used as controls. *APP*^KM670/671NL^/*PSEN1*^Δexon9^ *(APP/PS1)* mice and C57/BL6J WT mice were purchased from Huafukang Bioscience Co., Ltd. (Beijing, China). Mice were housed in groups of four with *ad libitum* access to standard food pellets and water on a 12/12h light/dark cycle. Experiments were approved by the Ethics Committee for Animal Experiments at The Affiliated Hospital of Jining Medical University.

### Morris Water Maze

Each group in this test contained six mice aged 2, 6, and 9 months. Testing was conducted using a standard 5-day regimen with a circular pool filled with opaque water by handlers who were blinded to grouping. During training sessions, which were conducted once a day for the first 5 days, a platform was placed 1 cm below the surface, and the mice were placed into the water in different quadrants facing the pool wall. If the mouse failed to locate the platform within 60 s, it was guided to the platform and allowed to stay on the platform for 15 s. Animal trajectories were recorded using a video-based image tracking system and ANY maze software (Global Biotech, Mount Laurel, NJ, United States).

### Immunofluorescence Analysis

At 24 h after the Morris water maze testing, three mice per group were deeply anesthetized with 1% carbrital and perfused transcardially with 0.9% saline, followed by cold 4% paraformaldehyde in 0.1 M phosphate-buffered saline (PBS, pH 7.4). Brains were dissected out and maintained overnight in 4% paraformaldehyde, cryopreserved in PBS containing 30% sucrose, then stored at −70°C until use. Brain sections (10 μm) were prepared and incubated for 12 h with one of the following antibodies: mouse monoclonal antibody against TYROBP (B-2, 1:100, cat# sc-166086, Santa Cruz Biotechnology, Dallas, TX, United States), mouse monoclonal antibody against tau (Tau5, 1:100, cat# ab80579, Abcam, Cambridge, MA, United States), mouse monoclonal antibody against Ser202/Thr205-phosphorylated tau (AT8, 1:100, cat# MN1020, Thermo Fisher Scientific, Waltham, MA, United States), mouse antibody against Aβ(1-16) (6E10, 1:100, cat# SIG-39320, Biolegend, San Diego, CA, United States), rabbit antibody against m6A (1:200, cat# A17924, ABclonal, Wuhan, Hubei, China) mixed with 2% BSA, 1 × DNase I Buffer (10 mM Tris-HCl, 2.5 mM MgCl2, 0.5 mM CaCl2), 25 U/mL DNase I (cat# 79254, Qiagen, Beverly, MA, United States), mouse monoclonal antibody against Iba1 (1:100, cat# ab283319, Abcam), rabbit monoclonal antibody against Iba1 (1:100, cat# ab178846, Abcam), mouse monoclonal antibody against GFAP (1:200, cat# CL488-60190, Proteintech Group, Chicago, IL, United States) and mouse monoclonal antibody against NeuN (1:200, cat# 66836-1-Ig, Proteintech Group). The sections were then incubated for 1 h with either Alexa Flour 488-conjugated goat anti-rabbit IgG (1:50, cat# SA00013-2, Proteintech Group) or Alexa Flour 568-conjugated goat anti-mouse IgG (1:1,000, cat# ab175473, Abcam). Images were acquired using an upright Zeiss microscope (Axio Imager.Z2, Carl Zeiss, Oberkochen, Germany), and analyzed using Image J (National Institutes of Health, Bethesda, MD, United States) (Schneider et al., 2012).

### RNA Isolation and Quantitative Real-Time PCR

Total RNA was isolated from each group of nine mice aged 6 months, then purified using TRIzol reagent (cat# 15596018, Invitrogen, Carlsbad, CA, United States). The amount and quality of the purified RNA were examined using the ND-1000 system (NanoDrop, Wilmington, DE, United States). Only RNA giving an absorbance ratio A_260_/A_280_ of 1.8–2.0 was used in further experiments. An aliquot of mRNA (1 μg per sample) was reverse-transcribed into cDNA using the SuperScript III First-StrandKit (cat# 18080051, Invitrogen), and 1 μL of cDNA (diluted 1:2) was used as template in quantitative PCR in the ChamQ™ Universal SYBR qPCR Master Mix (cat# Q711-02, Vazyme, Nanjing, Jiangsu, China). β-actin served as the internal control. Primer sequences were designed using the online Primer Blast tool (^1^ Supplementary Table 1). Levels of mRNA were expressed using the 2^–ΔΔCt^ method (Lv et al., 2016). Only genes associated with transcript Ct ≤ 30 were considered to be expressed.

### Western Blotting

Total protein was isolated from frozen hippocampi from groups of six mice aged 6 months using RIPA lysis buffer (Beyotime Biotechnology, Nanjing, China) containing PMSF (Beyotime Biotechnology). Lysates were left standing for 30 min, then centrifuged at 12,000 × *g* for 20 min at 4°C. Protein concentration was estimated using bicinchoninic acid (Beyotime Biotechnology), and equal amounts (30 μg) were separated by electrophoresis on precast 10% Bis-Tris gels (Bio-Rad Laboratories, Hercules, CA, United States), transferred to polyvinylidene difluoride membranes, and incubated with one of the following primary antibodies: rabbit antibody against TYROBP (B-2, 1:200, cat# sc-166086, Santa Cruz Biotechnology), rabbit antibody against METTL3 (1:1,000, cat# ab195352, Abcam), rabbit antibody against METTL14 (1:1,000, cat# A8530, ABclonal), rabbit antibody against WTAP (1:1,000, cat# 56501, Cell Signaling Technology, Danvers, MA, United States) and mouse antibody against GAPDH (1:50,000, cat# AC033, ABclonal). Secondary antibodies included horseradish peroxidase-conjugated goat anti-rabbit secondary IgG (1:5,000, cat# AS014, ABclonal) and goat anti-mouse IgG (1:5,000, cat# AS003, ABclonal). Antibody binding was visualized using enhanced chemiluminescence (cat# 32106, Thermo Fisher Scientific) and a Tanon 5200 imaging analysis system (Tanon Technology, Shanghai, China). Band intensities were analyzed using Image J.

### Enzyme-Linked Immunosorbent Assay

RIPA-soluble protein was isolated from the brains of groups of three to six mice aged 2, 6, and 9 months. Aβ levels were quantified using commercial ELISAs against Aβ40 (cat# MU30299, BIOSWAMP, Wuhan, Hubei, China) and Aβ42 (cat# MU30114, BIOSWAMP) according to the manufacturer’s protocols.

### Quantification of m6A Methylation

Levels of m6A methylation in total hippocampal RNA from groups of six mice aged 6 months were measured using a commercial kit (cat# ab185912, Abcam) according to the manufacturer’s instructions. Each sample contained 1,000 ng of total RNA. Absorbance was measured at 450 nm and converted to m6A levels using a standard curve.

### Methylated RNA Immunoprecipitation Sequencing

The MeRIP-Seq required at least 100 μg RNA in each sample; therefore, the RNAs of three mouse hippocampi (either WT or *Tyrobp*^–/–^) were pooled as one sample for MeRIP-Seq. The RNA was isolated as described above, and its integrity was assessed using a Bioanalyzer 2100 (Agilent, CA, United States) and denaturing agarose gel electrophoresis. RNA was used only if the RNA integrity number > 7.0. Poly(A) RNA was purified from 50 μg total RNA using oligo(dT)_25_ Dynabeads (cat# 61005, Thermo Fisher Scientific), and fragmented into small pieces at 86°C for 7 min using a Magnesium RNA Fragmentation Module (cat# e6150, New England Biolabs, Ipswich, MA, United States). The cleaved RNA fragments were incubated at 4°C for 2 h with an antibody against m6A (cat# 202003, Synaptic Systems, Göttingen, Niedersachsen, Germany) in 50 mM Tris-HCl, 750 mM NaCl and 0.5% Igepal CA-630. Immunoprecipitated RNA was reverse-transcribed into cDNA using SuperScript™ II Reverse Transcriptase (cat# 1896649, Invitrogen), which was then used as template to synthesize U-labeled second-strand DNA using *E. coli* DNA polymerase I (cat# m0209, New England Biolabs), RNase H (cat# m0297, New England Biolabs) and dUTP (cat# R0133, Thermo Fisher Scientific). The blunt ends of strands were extended with A bases for ligation to indexed adapters. Each adapter contained a T-base overhang to allow it to be ligated to the A-tailed DNA. Single- or dual-index adapters were ligated to the fragments, which were selected by size using AMPureXP beads. The ligated products were treated with a heat-labile UDG enzyme (cat# m0280, New England Biolabs), then amplified by PCR under the following conditions: initial denaturation at 95°C for 3 min; eight cycles of denaturation at 98°C for 15 s, annealing at 60°C for 15 s, and extension at 72°C for 30 s; then final extension at 72°C for 5 min. The average insert size for the final cDNA library was 300 ± 50 bp. The library was subjected to 2 × 150-bp paired-end sequencing (PE 150) on an illumineNovaseq™ 6000 (Illumina, San Diego, CA, United States).

### Bioinformatic Analysis

Fastp software^2^ (Chen et al., 2018), with its default parameters, was used to remove adapter contamination and low-quality reads, defined as Q ≤ 10. Fastp was also used to verify sequence quality of the input and immunoprecipitated samples. We used HISAT2^3^ (Kim et al., 2015) to map the reads to the *Mus musculus* genome (version: v96). Mapped reads of immunoprecipitated and input libraries were analyzed using the exomePeak package in R^4^ (Meng et al., 2014), which identified m6A peaks using the bed or bigwig format. Output was visualized using IGV software^5^ (Robinson et al., 2011). MEME^6^ (Bailey et al., 2009) and HOMER^7^ were used to identify *de novo* and known motifs, followed by localization of the motif with respect to the peak summit.

Peaks were annotated based on intersection with gene architecture using the ChIPseeker package in R^8^ (Yu et al., 2015). The expression levels of all mRNAs in input libraries were assessed using StringTie^9^. FPKM was calculated as total exon fragments/mapped reads (millions) × exon length (kB). The mRNAs differentially expressed between *Tyrobp*^–/–^ and WT mice were defined as those showing fold change ≥ 2 or ≤ −2 and *P* < 0.05 based on the edgeR package in R^10^ (Robinson et al., 2010).

### Statistical Analysis

All statistical analyses were conducted using GraphPad Prism (version 8.0, Graphpad, San Diego, CA, United States). Data were presented as mean ± SEM. Pairwise comparisons were assessed for significance using Student’s *t* test for independent samples. Differences in the Morris water maze test were assessed using two-way ANOVA for repeated measures, followed by Tukey’s *post hoc* test. Differences in gene expression profiles were assessed in terms of fold change. *P* < 0.05 were considered statistically significant.

## Results

### Learning and Memory Deficits in *Tyrobp^–/–^* Mice

**FIGURE 1:**
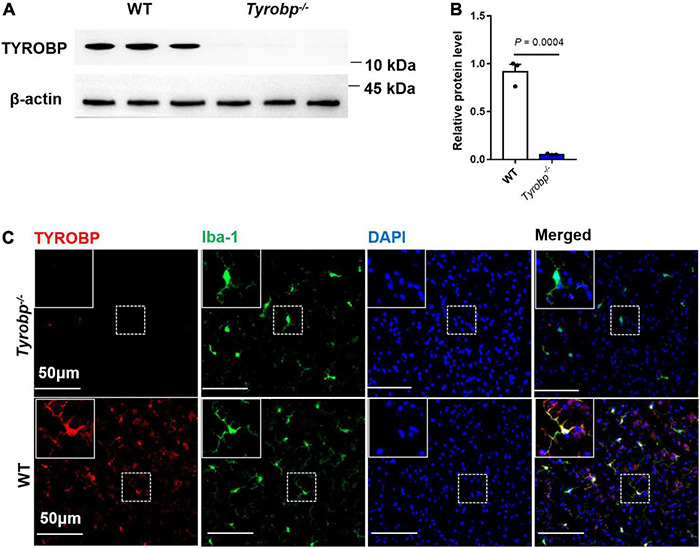
Western blotting and immunofluorescence analysis of wild-type (WT) and *Tyrobp^–/–^* mice. **(A,B)** Western blotting and quantification of TYROBP in WT and *Tyrobp^–/–^* mice. **(C)** Immunofluorescence analysis of WT and *Tyrobp^–/–^* mice. Data in panel **(B)** are mean ± SEM. Statistical significance was calculated using Student’s *t* test.

*Tyrobp^–/–^* mice showed extremely low expression of TYROBP, whereas WT animals showed abundant protein, especially in microglia (Figures 1A–C).

**FIGURE 2:**
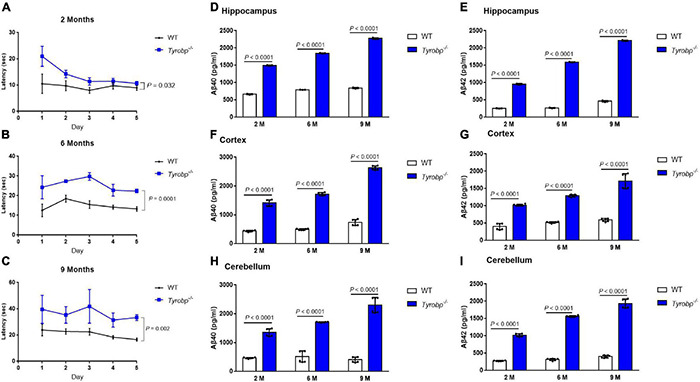
Performance in the Morris water maze and levels of soluble Aβ40 and Aβ42 in WT and *Tyrobp^–/–^* mice. **(A–C)** Escape latency of WT and *Tyrobp^–/–^* mice aged 2, 6, or 9 months. **(D,E)** Levels of soluble Aβ40 and Aβ42 in hippocampus of WT and *Tyrobp^–/–^* mice aged 2, 6, or 9 months (M), as detected by ELISA. **(F,G)** Levels of soluble Aβ40 and Aβ42 in cortex of WT and *Tyrobp^–/–^* mice aged 2, 6, or 9 months (M), as detected by ELISA. **(H,I)** Levels of soluble Aβ40 and Aβ42 in cerebellum of WT and *Tyrobp^–/–^* mice aged 2, 6, or 9 months (M), as detected by ELISA. Data are mean ± SEM (six mice per group). Differences were assessed for significance using two-way ANOVA for repeated measures, followed by Tukey’s *post hoc* test.

On day 1 in the Morris water maze, escape latency was higher for *Tyrobp^–/–^* mice than for WT animals aged 2 months (20.95 ± 3.31 vs. 10.51 ± 3.21 s, *P* = 0.008), 6 months (24.27 ± 4.80 vs. 12.54 ± 2.78 s, *P* = 0.019) or 9 months (39.63 ± 9.33 vs. 23.90 ± 3.86 s, *P* = 0.036; Figures 2A–C). Similar results were observed on day 5 among animals aged 2 months (10.65 ± 0.61 vs. 8.97 ± 0.81 s, *P* = 0.032), 6 months (22.38 ± 0.77 vs. 13.27 ± 0.99 s, *P* = 0.0001) or 9 months (33.39 ± 1.93 vs. 16.58 ± 1.02 s, *P* = 0.002; Figures 2A–C). In contrast, *Tyrobp^–/–^* and WT mice did not differ significantly in swimming speed (data not shown). *Tyrobp^–/–^* mice at all three ages showed higher levels of soluble Aβ40 and Aβ42 than WT mice in the hippocampus, cortex and cerebellum (Figures 2D–I).

### Higher Levels of Total Tau, Ser202/Thr205-Phosphorylated Tau and Aβ in *Tyrobp^–/–^* Mice

**FIGURE 3:**
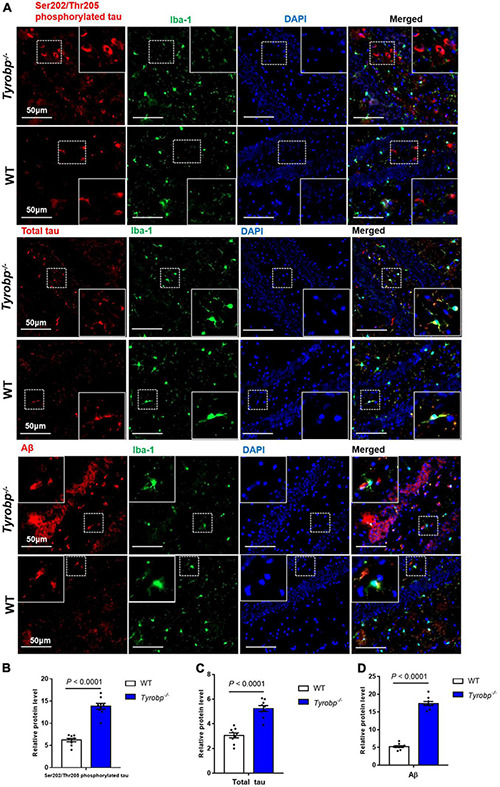
Numbers of hippocampal cells immunopositive for total tau, Ser202/Thr205-phosphorylated tau, and Aβ. Tissues from hippocampus of wild-type (WT) and *Tyrobp^–/–^* mice aged 6 months were immunostained for total tau (tau-5), Ser202/Thr205-phosphorylated tau (AT8), and Aβ (6E10). **(A)** Representative micrographs. **(B–D)** Quantification of total tau, Ser202/Thr205-phosphorylated tau, and Aβ. Data are mean ± SEM from three independent experiments (three mice per group). Differences were assessed for significance using Student’s *t* test.

Given that *Tyrobp^–/–^* mice showed abnormal behavior and elevated levels of soluble Aβ40 and Aβ42 at 2, 6, and 9 months, we used six-month-old animals in subsequent experiments. *Tyrobp^–/–^* mice showed significantly higher hippocampal levels of total tau (5.23 ± 0.24 vs. 3.06 ± 0.20%, *P* < 0.0001), Ser202/Thr205-phosphorylated tau (13.82 ± 0.64 vs. 6.20 ± 0.37%, *P* < 0.0001) and Aβ (17.40 ± 0.56 vs. 5.24 ± 0.28%, *P* < 0.0001; Figure 3). Similar results were observed in the cortex: total tau, 8.82 ± 0.45 vs. 4.82 ± 0.32%, *P* < 0.0001; Ser202/Thr205-phosphorylated tau, 12.75 ± 0.48 vs. 3.71 ± 0.45%, *P* < 0.0001; and Aβ, 15.39 ± 1.92 vs. 5.09 ± 1.10%, *P* < 0.0001 (Supplementary Figure 1). Similar results were also observed in the cerebellum: total tau, 7.91 ± 0.31 vs. 4.76 ± 0.27%, *P* < 0.0001; Ser202/Thr205-phosphorylated tau, 7.98 ± 0.53 vs. 3.68 ± 0.28%, *P* < 0.0001; and Aβ, 7.27 ± 0.43 vs. 3.32 ± 0.24%, *P* < 0.0001 (Supplementary Figure 2).

**FIGURE 4:**
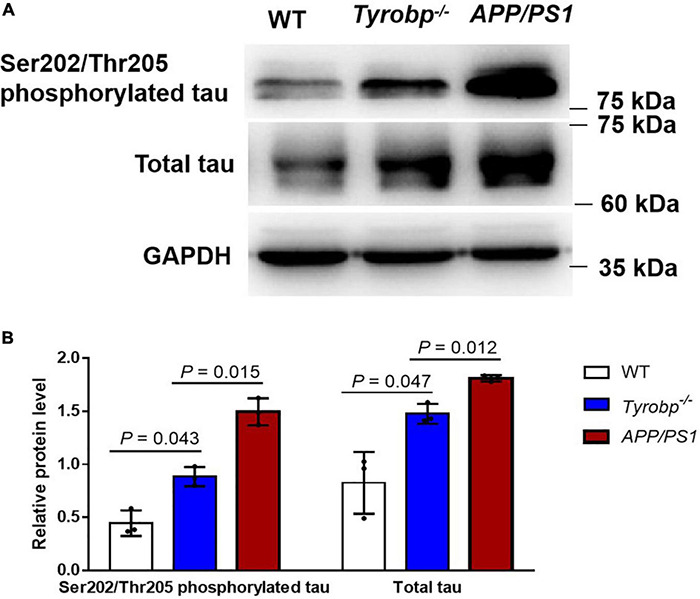
Levels of total tau and Ser202/Thr205-phosphorylated tau in WT, *Tyrobp^–/–^* and *APP/PS1* mice. **(A)** Western blot analysis. **(B)** Quantification of total tau and Ser202/Thr205-phosphorylated tau. Data are mean ± SEM from three independent experiments (three mice per group). Differences were assessed for significance using Student’s *t* test.

To benchmark the phenotype of *Tyrobp^–/–^* mice against an AD phenotype, we compared hippocampal levels of total tau and Ser202/Thr205-phosphorylated tau between *Tyrobp^–/–^* and *APP/PS1* mice, all 15 months old. *Tyrobp^–/–^* mice showed higher levels of Ser202/Thr205-phosphorylated tau (0.45 ± 0.10 vs. 0.88 ± 0.07, *P* = 0.043) and total tau (0.79 ± 0.22 vs. 1.51 ± 0.07, *P* = 0.047) than WT animals. However, *Tyrobp^–/–^* mice showed lower levels of Ser202/Thr205-phosphorylated tau (0.79 ± 0.22 vs. 1.49 ± 0.10, *P* = 0.015) and total tau (1.51 ± 0.07 vs. 1.81 ± 0.02, *P* = 0.012) than the *APP/PS1* mice (Figure 4).

### Reduced m6A Methyltransferases in the Hippocampus of *Tyrobp^–/–^* Mice

**FIGURE 5:**
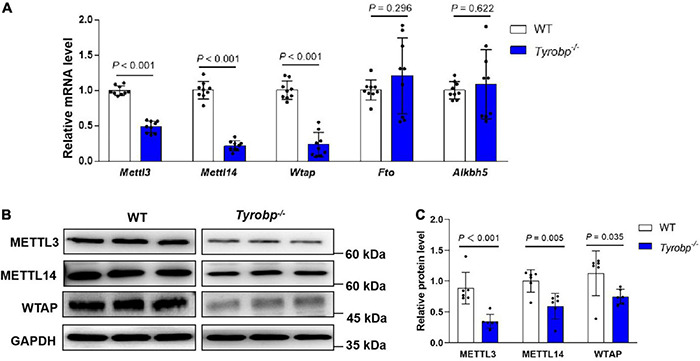
Reduced m6A RNA methyltransferases in the hippocampus of *Tyrobp^–/–^* mice. **(A)** Relative levels of *Mettl3*, *Mettl14*, and *Wtap* mRNAs encoding methyltransferases and of *Fto* and *Alkbh5* mRNAs encoding demethylases. **(B,C)** Western blotting and quantification of METTL3, METTL14 and WTAP in the hippocampus. Data are mean ± SEM (six or nine mice per group). Differences were assessed for significance using Student’s *t* test.

*Tyrobp^–/–^* mice contained significantly lower hippocampal levels of *Mettl3*, *Mettl14*, and *Wtap* mRNAs, which encode methyltransferases (*P* < 0.001, Figure 5A). These mRNA results were verified at the protein level by western blotting (Figures 5B,C). In contrast, the two types of animals did not differ significantly in expression of the *Fto* or *Alkbh5* genes encoding demethylases.

The global m6A RNA methylation level in hippocampal was significantly lower in six-month-old *Tyrobp^–/–^* mice than in age-matched WT animals (0.0357 ± 0.00008 vs. 0.0529 ± 0.00005%, *P* = 0.005), (Supplementary Figure 3A). Similarly, based on immunofluorescence staining, the m6A RNA methylation were significantly lower in six-month-old *Tyrobp^–/–^* mice than in age-matched WT animals (12.12 ± 1.69 vs. 5.54 ± 0.81, *P* = 0.024) (Supplementary Figure 3B), and the decrease in m6A methylation occurred in microglia (1.14 ± 0.12 vs. 0.63 ± 0.06, *P* = 0.020) (Supplementary Figures 3C,D), astrocytes (4.27 ± 0.39 vs. 2.76 ± 0.30, *P* = 0.037) (Supplementary Figures 3E,F) and neurons (10.17 ± 1.20 vs. 4.78 ± 0.55, *P* = 0.015) (Supplementary Figures 3G,H).

### Altered m6A RNA Methylation Patterns in *Tyrobp^–/–^* Mice

**FIGURE 6:**
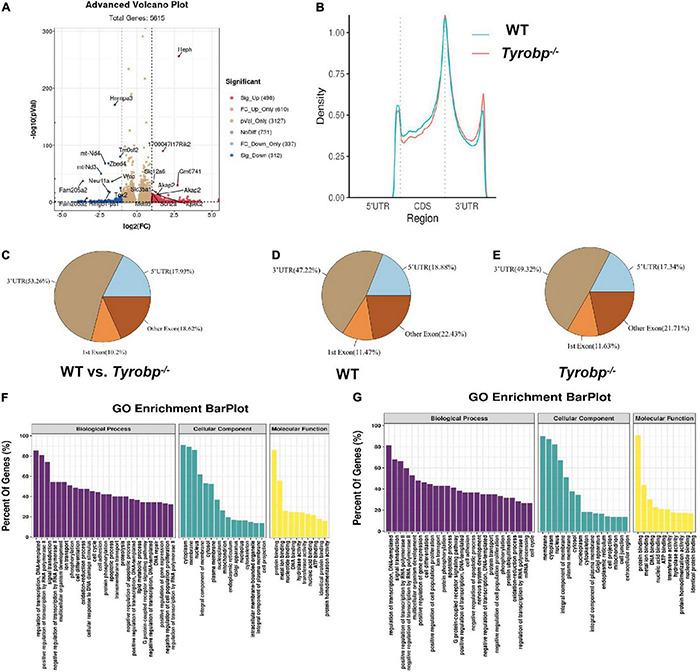
Characteristics of m6A methylation in the hippocampus of 6-month-old *Tyrobp^–/–^* mice. **(A)** Volcano plots showing m6A peaks differing significantly between *Tyrobp^–/–^* and wild-type (WT) mice. **(B)** Average distribution of m6A peaks along transcripts in the hippocampus. **(C–E)** Pie charts showing the distribution of m6A peaks. **(F)** Major Gene Ontology terms for transcripts whose m6A methylation was upregulated in *Tyrobp^–/–^* mice. **(G)** Major Gene Ontology terms for transcripts whose m6A methylation was downregulated in *Tyrobp^–/–^* mice. Up- or downregulation was defined, respectively, as fold change ≥ 2 or fold change ≤ –2 combined with *P* < 0.05. Nine mice were used in each group.

The original data, which were deposited in the GEO database under accession number GSE179827, were of generally high quality, with > 97% of reads meeting the Q20 criterion and > 92% of reads exceeding the Q30 criterion (Supplementary Table 2). *Tyrobp^–/–^* mice showed 810 m6A peaks differing significantly from WT animals (Supplementary Table 3), of which 498 peaks were significantly higher and 312 significantly lower in *Tyrobp^–/–^* mice (Figure 6A). In *Tyrobp^–/–^* and WT mice, the m6A peaks were enriched mainly near stop codons and 3′-untranslated regions (Figure 6B), and the peaks differing significantly between the two types of mice occurred most often in the 3′-untranslated region (53.26%), followed by other exons (18.62%), 5′-untranslated regions (17.93%), and first exons (10.2%) (Figure 6C). Compared to WT animals, *Tyrobp^–/–^* mice showed higher proportions of m6A peaks in 3′-untranslated regions (49.32 vs. 47.22%) and first exons (11.63 vs. 11.47%), but lower proportions in 5′-untranslated regions (17.34 vs. 18.88%) and other exons (21.71 vs. 22.43%) (Figures 6D,E).

In Gene Ontology (GO) analysis, the upregulated peaks were significantly associated with the following biological processes: regulation of DNA-templated transcription, positive regulation of transcription by RNA polymerase II and signal transduction. The upregulated peaks were associated with the cellular components of cytoplasm, membrane and nucleus; and they were associated with the molecular functions of protein binding, metal ion binding and nucleotide binding (Figure 6F).

The downregulated peaks, in contrast, were associated mainly with the biological processes of regulation of DNA-templated transcription, signal transduction, and positive regulation of transcription by RNA polymerase II (Figure 6G). Downregulated peaks were also associated with the cellular components of membrane, cytoplasm and nucleus; and they were associated with the molecular functions of protein binding, metal ion binding and DNA binding.

### Altered Hippocampal Gene Expression in *Tyrobp^–/–^* Mice

**FIGURE 7:**
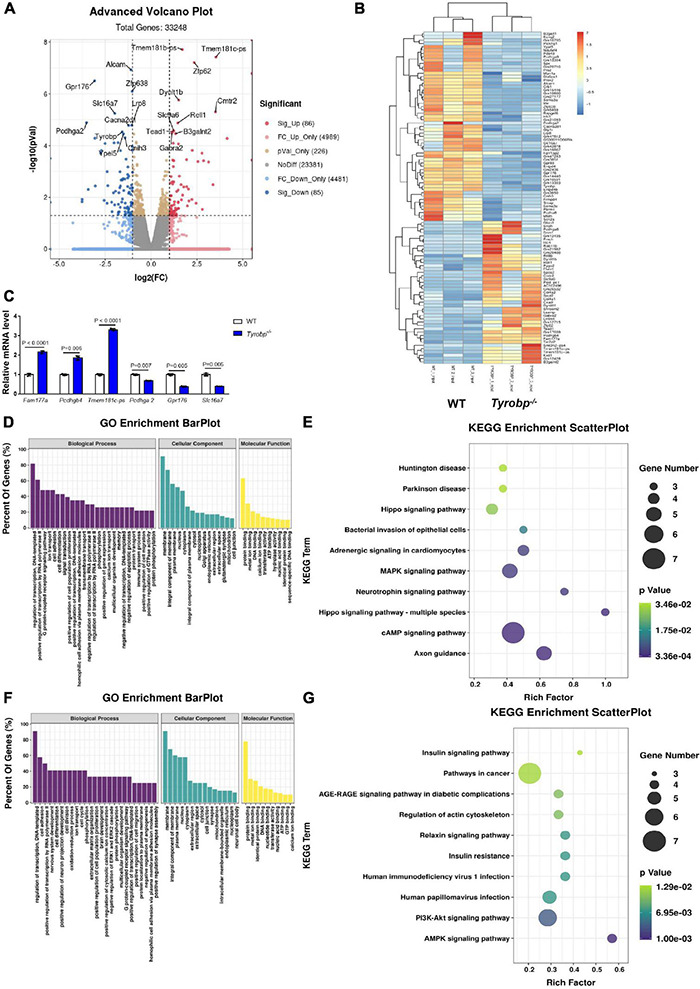
Volcano plots, heatmaps, Gene Ontology (GO) terms and Kyoto Encyclopedia of Genes and Genomes (KEGG) pathways enriched in genes differentially expressed between wild-type (WT) and *Tyrobp^–/–^* mice. **(A)** Volcano plots and **(B)** heatmap showing genes and transcripts differentially expressed between WT and *Tyrobp^–/–^* mice. **(C)** Quantitative real-time PCR detection of six genes chosen from the methylated RNA immunoprecipitation sequencing data. **(D,E)** Major GO terms and KEGG pathways were analyzed for genes upregulated by *Tyrobp* knockout. **(F,G)** Major GO terms and KEGG pathways were analyzed for genes downregulated by *Tyrobp* knockout. Up- or downregulation was defined, respectively, as fold change ≥ 2 or fold change ≤ –2 combined with *P* < 0.05. Nine mice were used in each group.

RNA sequencing data showed that 86 genes were upregulated in *Tyrobp^–/–^* mice relative to WT controls, while 85 genes were down-regulated (Supplementary Table 4). The top five upregulated genes were *Fam177a, Pmch, Pcdhgb4, Hcrt*, and *Tmem181c-ps*, and the top five downregulated genes were *Mgam, Pcdhga2, Gpr176, Pcdhga9*, and *Proz* (Figures 7A,B). The altered expression of three upregulated genes (*Fam177a*, *Pcdhgb4* and *Tmem181c-ps*) and three downregulated genes (*Pcdhga2*, *Gpr176* and *Slc16a7*) was verified using quantitative real-time PCR (Figure 7C).

The major GO terms and KEGG pathways involving upregulated genes are shown in Figures 7D,E. Upregulated KEGG pathways included the cAMP signaling pathway, axon guidance and MAPK signaling pathway (Figure 7E). The major GO terms and KEGG pathways involving downregulated genes are shown in Figures 7F,G. Downregulated KEGG pathways included the AMPK signaling pathway, PI3K-Akt signaling pathway, and human papillomavirus infection (Figure 7G).

### Correlation of Altered m6A RNA Methylation With Altered Gene Expression in the Hippocampus of *Tyrobp^–/–^* Mice

**FIGURE 8:**
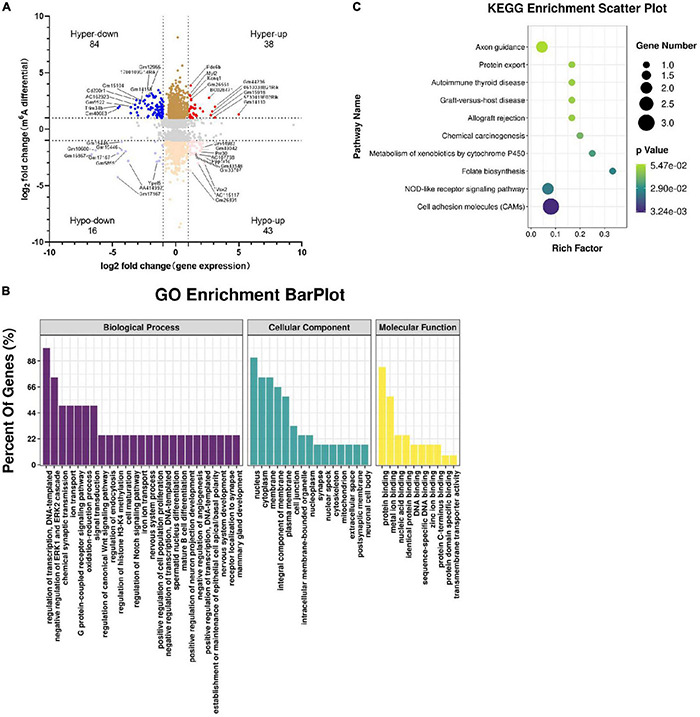
Combined analyses of m6A-RIP-seq and RNA-seq data. Genes whose expression and m6A methylation differed significantly between *Tyrobp^–/–^* and wild-type mice were analyzed in terms of their **(A)** four-quadrant distribution (see Results), **(B)** major GO terms, and **(C)** top 10 KEGG pathways. Fold change ≥ 2 or fold change ≤ –2 and *P* < 0.05, nine mice were used in each group.

We identified genes whose m6A methylation at the RNA level and whose gene expression were altered (Supplementary Table 5), leading to four groups (Figure 8A): hypermethylation and upregulation, 38 genes; hypomethylation and downregulation, 16 genes; hypomethylation and upregulation, 43 genes; and hypermethylation and downregulation, 84 genes.

In GO analysis, the top three biological processes were regulation of DNA-templated transcription, negative regulation of ERK1 and ERK2 cascades, and chemical synaptic transmission. The top three cellular components were nucleus, cytoplasm, and membrane, while the top three molecular functions were protein binding, metal ion binding, and nucleic acid binding (Figure 8B). The KEGG analysis enriched for the overlap genes were cell adhesion molecules, NOD-like receptor (NLR) signaling and folate biosynthesis (Figure 8C).

Cell adhesion molecules of interest included Cldn19, H2-M5 and Alcam. In *Tyrobp^–/–^* mice, these genes were m6A-hypomethylated and upregulated. The NLR signaling pathway includes *Nlrp6* and *Pstpip1*. In *Tyrobp^–/–^* mice, the *Nlrp6* gene was m6A-hypermethylated and downregulated, while the *Pstpip1* gene was m6A-hypomethylated and upregulated.

## Discussion

Here we demonstrate AD-like histopathology and behavioral deficits in *Tyrobp^–/–^* mice, which were linked to downregulation of the methyltransferases METTL3, METTL14, and WTAP. These findings suggest that TYROBP deficiency may associate with an altered m6A epitranscriptome in hippocampus as well as altered expression of a variety of genes and signaling pathways involved in cognitive function.

*Tyrobp^–/–^* mice mimic central features of NHD and AD, most notably learning and memory deficits (Cui et al., 2021). *Tyrobp^–/–^* mice in our study showed elevated levels of total tau, Ser202/Thr205-phosphorylated tau and Aβ in the hippocampus, cortex and cerebellum. These elevated hippocampal levels have also been observed in NHD patients (Satoh et al., 2018). NHD patients carrying the Q33X mutation in the *TREM2* gene show AD-like lesions, including Aβ deposition and neurofibrillary changes (Maderna et al., 2021). However, whether deficiency of *TYROBP* contributes to, or protects against neurodegenerative disease, remains controversial. Deleting the *TYROBP* gene from *APP*^KM670/671NL^/*PSEN1*^Δexon9^ mice mitigated behavioral and electrophysiological deficits, without affecting the total number of Iba1-positive microglia in cortex or hippocampus (Haure-Mirande et al., 2017, 2019). Deleting the *TYROBP* gene from *MAPT^P301S^* mice also mitigated clinical and electrophysiological deficits, while paradoxically increasing tau hyperphosphorylation and spreading (Audrain et al., 2019). Overexpression of TYROBP in microglia of mice can decrease amyloid burden but increase tau phosphorylation in an *APP/PSEN1* or *MAPT^P301S^* background (Audrain et al., 2021). These considerations lead us to speculate that each particular state of microglial activation is associated with an optimal level of TYROBP expression.

The m6A methylation is the most frequent reversible post-transcriptional methylation of mammalian mRNA. The level of m6A methylation increases in the mouse brain during adulthood (Meyer et al., 2012), and it helps regulate long-term memory formation (Zhang et al., 2018), facilitating hippocampus-dependent learning and memory through the YTHDF1 protein (Shi et al., 2018). AD patients show decreased m6A methylation in neurons of the hippocampus and cortex, but higher methylation in GFAP-positive astrocytes and some Iba1-positive microglia (Zhao et al., 2021). The *APP/PS1* mouse model of AD, in contrast, shows elevated m6A methylation, but the immunofluorescence analysis were not done (Han et al., 2020). *Tyrobp^–/–^* mice in our study showed reduced m6A methylation in microglia, astrocytes and in neurons. However, the immunofluorescence cannot report differentially on ribosomal RNA and mRNA, since they colocalize, an mRNA-enriched ELISA is needed in the future. Further investigation is also needed to clarify whether NHD and AD involve similar changes in m6A methylation, and to explore how these changes correlate with the pathological hallmarks of the two diseases.

In our study, METTL3, METTL14, and WTAP were downregulated in *Tyrobp^–/–^* mice, and the same proteins were downregulated in hippocampus and cortex of AD patients (Zhao et al., 2021). METTL3 plays a key role in m6A methylation, and depleting METTL3 from the hippocampus of C57BL/6 mice compromises memory consolidation (Zhang et al., 2018) and triggers extensive synaptic loss, neuronal death, oxidative stress and aberrant cell cycling (Zhao et al., 2021). Other studies have linked AD in humans to accumulation of insoluble METTL3 in hippocampus (Huang et al., 2020), or even to upregulation of METTL3 in the cortex and hippocampus of one mouse model of AD (Han et al., 2020). These conflicting results highlight the need for future studies to clarify how altered m6A methyltransferase activity may contribute to neurological diseases.

Knocking out TYROBP in our mice did not alter expression of FTO or ALKBH5, consistent with a report of normal FTO and ALKBH5 levels in soluble and insoluble hippocampal fractions from AD patients (Huang et al., 2020). Other studies, however, have reported downregulated FTO in the hippocampus of AD patients and an AD mouse model (Han et al., 2020; Zhao et al., 2021), as well as in the cortex of a rat model for traumatic brain injury (Yu et al., 2020). These discrepancies may depend on the animal species, pathological state, disease stage, and brain fraction (soluble or insoluble) being studied.

Our study appears to report the first analysis of a relationship between differentially expressed m6A peaks and genes in NHD or AD. We found many of the differentially expressed m6A peaks to be related with cancer, including peaks affecting the genes *Trim58*, *Fam83f*, *Whrn*, and *Pde6b*. Among the differentially expressed genes known to be relevant to neuronal functions, the *Pcdhs* cluster, including *Pcdha* and *Pcdhg*, are highly expressed in the brain and help guide neurons during brain development, neuronal differentiation, and synaptogenesis (Shimojima et al., 2011). In *Tyrobp^–/–^* mice, *Pcdhgb4* and *Pcdhga8* were upregulated, while *Pcdhga2, Pcdhga9* and *Pcdhgb8* were downregulated, suggesting that *Pcdhg* gene expression might have some relation with TYROBP. *Fam177a* was upregulated in our *Tyrobp^–/–^* mice, and downregulation of this gene impairs the transduction of TLR4 signaling; inhibits the phosphorylation of NF-κB, Akt, Erk1/2 and JNK; and downregulates TLR4, MyD88, TRAF6, and TRIF (Chen et al., 2021). The potential implication of *Fam177a* in AD requires further investigation.

Our *Tyrobp^–/–^* mice showed dysregulation of several pathways, including signaling pathways involving cAMP, MAPK, AMPK, and PI3K-Akt. TYROBP may interact with SHIP1 and DOK3 to inhibit Syk, PI3K, and ERK, as well as signaling involving Toll-like receptors in macrophages (Xing et al., 2015; Hamerman et al., 2016). PI3K activates Akt to inhibit mitogen-activated protein kinase kinase kinase, which in turn inhibits Toll-like receptors (Hamerman et al., 2009). Analysis of the overlap between m6A methylation and gene expression identified several pathways, such as the NLR signaling pathway. Unlike Toll-like receptors, NOD-like receptors are intracellular proteins that scan the cytoplasm for the presence of intracellular invaders. Future studies are needed to examine whether NLRs also contribute to NHD.

Our *Tyrobp^–/–^* mice showed dysregulation of pathways involving cell adhesion molecules. Neural cell adhesion molecules are involved in memory formation and consolidation (Welzl and Stork, 2003), and they also regulate synapse formation, maintenance and function (Thalhammer and Cingolani, 2014). Levels and function of synaptic cell adhesion molecules are altered in brain tissues, cerebrospinal fluid, and sera in AD patients (Leshchyns’ka and Sytnyk, 2016). Our results provide a hint of a relationship between cell adhesion molecules and NHD, so the potential role of these molecules in memory impairment should be explored.

In summary, our study demonstrates that *Tyrobp^–/–^* mice share behavioral and pathological features of mouse models of AD. Knocking out TYROBP reduced m6A methyltransferases components METTL3, METTL14, and WTAP. These and other observed alterations in m6A methylation profiles may provide clues to the pathogenesis of NHD, AD and other diseases related to the *Tyrobp* gene. Further study is necessary to investigate how decreasing m6A methylation mediates the histopathological and behavioral deficits of *Tyrobp^–/–^* mice.

## Data Availability Statement

The datasets presented in this study can be found in online repositories. The names of the repository/repositories and accession number(s) can be found below: Gene Expression Omnibus accession: GSE179827, available at: https://www.ncbi.nlm.nih.gov/geo/query/acc.cgi?acc=GSE179827.

## Ethics Statement

The animal study was reviewed and approved by Ethical Committee for Animal Experiments at The Affiliated Hospital of Jining Medical University.

## Author Contributions

ZL and YH designed the study. TX, RL, and YL conducted the experiments. ZL, TX, DZ, WL, RL, and YY analyzed the data. ZL and TX wrote the manuscript. All authors approved the manuscript for publication.

## Conflict of Interest

The authors declare that the research was conducted in the absence of any commercial or financial relationships that could be construed as a potential conflict of interest.

## Publisher’s Note

All claims expressed in this article are solely those of the authors and do not necessarily represent those of their affiliated organizations, or those of the publisher, the editors and the reviewers. Any product that may be evaluated in this article, or claim that may be made by its manufacturer, is not guaranteed or endorsed by the publisher.

## Footnotes

1
https://www.ncbi.nlm.nih.gov/tools/primer-blast

2
https://github.com/OpenGene/fastp

3
http://daehwankimlab.github.io/hisat2

4
https://bioconductor.org/packages/exomePeak

5
https://www.igv.org

6
https://meme-suite.org

7
http://homer.ucsd.edu/homer/motif

8
https://bioconductor.org/packages/ChIPseeker

9
https://ccb.jhu.edu/software/stringtie

10
https://bioconductor.org/packages/edgeR

## Supplementary Material

Supplementary Figure 1 Numbers of cells immunopositive for total tau, Ser202/Thr205-phosphorylated tau, and Aβ in cortex. Cortical tissues were immunostained for total tau (tau-5), Ser202/Thr205-phosphorylated tau (AT8), and Aβ (6E10) in 6-month-old *Tyrobp^–/–^* and wild-type (WT) mice. **(A)** Representative micrographs. **(B–D)** Quantification of total tau, Ser202/Thr205-phosphorylated tau, and Aβ. Data are mean ± SEM from three independent experiments (three mice per group). Differences were assessed for significance using Student’s *t* test.

Supplementary Figure 2 Numbers of cells immunopositive for total tau, Ser202/Thr205-phosphorylated tau, and Aβ in cerebellum. Cerebellum tissues were immunostained for total tau (tau-5), Ser202/Thr205-phosphorylated tau (AT8), and Aβ (6E10) in 6-month-old *Tyrobp^–/–^* and wild-type (WT) mice. **(A)** Representative micrographs. **(B–D)** Quantification of total tau, Ser202/Thr205-phosphorylated tau, and Aβ. Data are mean ± SEM from three independent experiments (three mice per group). Differences were assessed for significance using Student’s *t* test.

Supplementary Figure 3 Reduced m6A RNA methylation in hippocampus of *Tyrobp^–/–^* mice. **(A)** Quantification of total m6A RNA methylation in *Tyrobp^–/–^* mice and wild-type (WT) mice. **(B)** Quantification of total m6A RNA methylation in *Tyrobp^–/–^* mice and wild-type (WT) mice, based on immunofluorescence staining. **(C,D)** Immunofluorescence staining and quantification analysis of m6A RNA methylation with microglia marker IBA1 in hippocampal tissues from *Tyrobp^–/–^* mice and WT mice. **(E,F)** Immunofluorescence staining and quantification analysis of m6A RNA methylation with astrocytes marker GFAP in hippocampal tissues from *Tyrobp^–/–^* mice and WT mice. **(G,H)** Immunofluorescence staining and quantification analysis of m6A RNA methylation with neuron marker NeuN in hippocampal tissues from *Tyrobp^–/–^* mice and WT mice. Data are mean ± SEM (three to six mice per group). Differences were assessed for significance using Student’s *t* test.

Supplementary Table 1 Sequences of primers used for RT-qPCR analysis of mRNA levels.

Supplementary Table 2 Information of quality control of the raw data.

Supplementary Table 3 Comparison of m6A RNA methylation in *Tyrobp^–/–^* and wild-type (WT) mice.

Supplementary Table 4 Genes detected in *Tyrobp^–/–^* and wild-type (WT) mice.

Supplementary Table 5 Gene expression and m6A peaks overlapping between *Tyrobp^–/–^* and wild-type (WT) mice.
