# Supplementary figures and images for: Downregulation of m6A Methyltransferase in the Hippocampus of Tyrobp–/– Mice and Implications for Learning and Memory Deficits
Source: Front Neurosci. 2022 Mar 21;16:739201. doi: 10.3389/fnins.2022.739201 (PMC8978996; doi:10.3389/fnins.2022.739201)

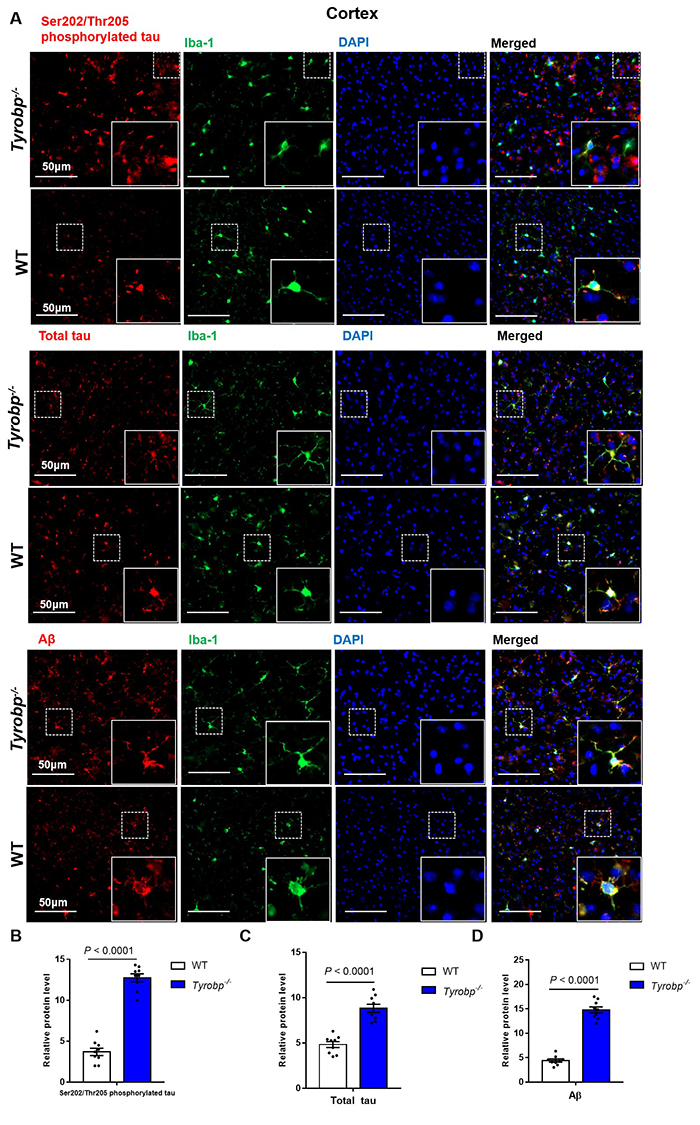

Supplement: Supplementary Figure 1 — Numbers of cells immunopositive for total tau, Ser202/Thr205-phosphorylated tau, and Aβ in cortex. Cortical tissues were immunostained for total tau (tau-5), Ser202/Thr205-phosphorylated tau (AT8), and Aβ (6E10) in 6-month-old Tyrobp–/– and wild-type (WT) mice. (A) Representative micrographs. (B–D) Quantification of total tau, Ser202/Thr205-phosphorylated tau, and Aβ. Data are mean ± SEM from three independent experiments (three mice per group). Differences were assessed for significance using Student’s t test. [file Image_1.JPEG]

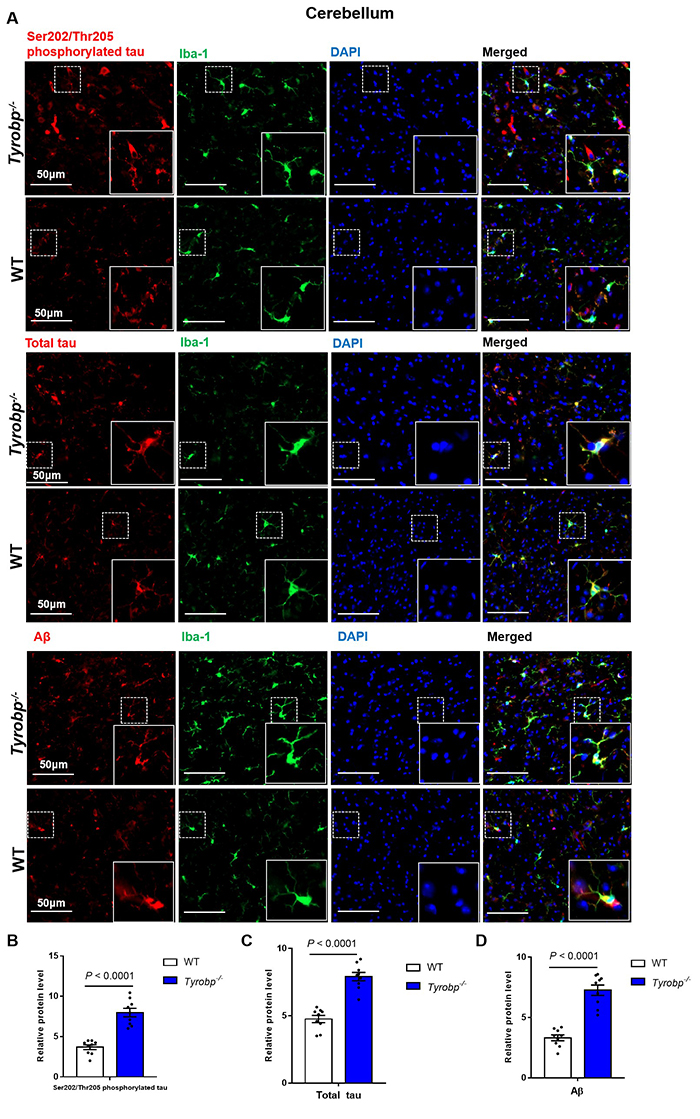

Supplement: Supplementary Figure 2 — Numbers of cells immunopositive for total tau, Ser202/Thr205-phosphorylated tau, and Aβ in cerebellum. Cerebellum tissues were immunostained for total tau (tau-5), Ser202/Thr205-phosphorylated tau (AT8), and Aβ (6E10) in 6-month-old Tyrobp–/– and wild-type (WT) mice. (A) Representative micrographs. (B–D) Quantification of total tau, Ser202/Thr205-phosphorylated tau, and Aβ. Data are mean ± SEM from three independent experiments (three mice per group). Differences were assessed for significance using Student’s t test. [file Image_2.JPEG]

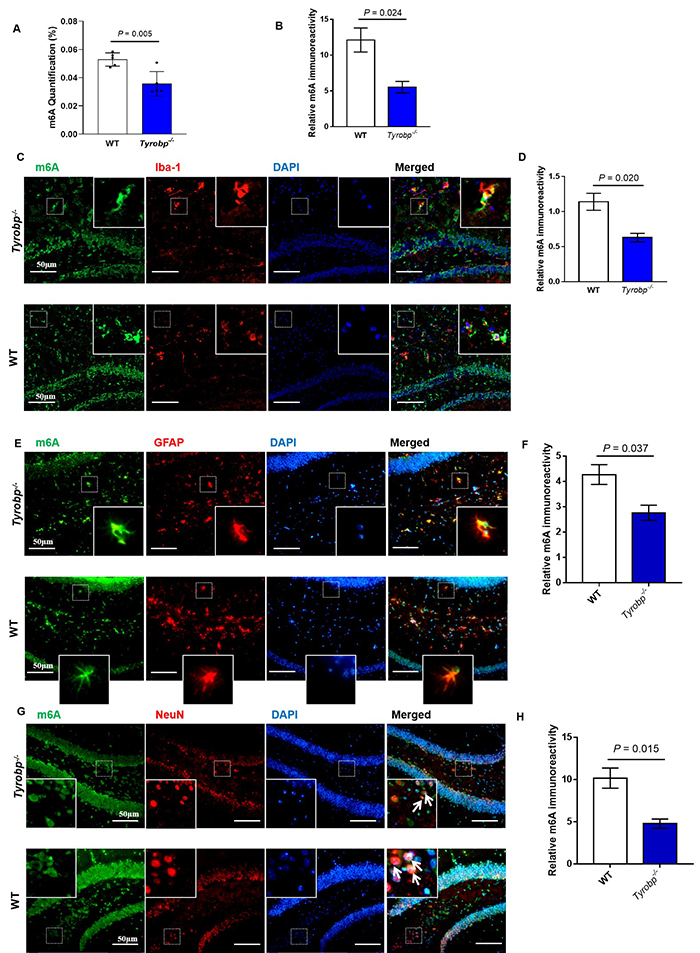

Supplement: Supplementary Figure 3 — Reduced m6A RNA methylation in hippocampus of Tyrobp–/– mice. (A) Quantification of total m6A RNA methylation in Tyrobp–/– mice and wild-type (WT) mice. (B) Quantification of total m6A RNA methylation in Tyrobp–/– mice and wild-type (WT) mice, based on immunofluorescence staining. (C,D) Immunofluorescence staining and quantification analysis of m6A RNA methylation with microglia marker IBA1 in hippocampal tissues from Tyrobp–/– mice and WT mice. (E,F) Immunofluorescence staining and quantification analysis of m6A RNA methylation with astrocytes marker GFAP in hippocampal tissues from Tyrobp–/– mice and WT mice. (G,H) Immunofluorescence staining and quantification analysis of m6A RNA methylation with neuron marker NeuN in hippocampal tissues from Tyrobp–/– mice and WT mice. Data are mean ± SEM (three to six mice per group). Differences were assessed for significance using Student’s t test. [file Image_3.JPEG]
