# Supplementary material for: Downregulation of m6A Methyltransferase in the Hippocampus of Tyrobp–/– Mice and Implications for Learning and Memory Deficits
Source: Front Neurosci. 2022 Mar 21;16:739201. doi: 10.3389/fnins.2022.739201 (PMC8978996; doi:10.3389/fnins.2022.739201)
Supplement: Supplementary Table 1 — Sequences of primers used for RT-qPCR analysis of mRNA levels. [file Table_1.DOCX]

**Supplementary table 1.** Sequences of primers used for RT-qPCR analysis of mRNA levels.

| Gene | Sequence | Product size (bp) |
| --- | --- | --- |
| *Mwttl3* | Forward: 5’ CGCTGCCTCCGATGTTGATCTG 3’ | 85 |
| *Mettl14*  *Wtap*  *Fto*  *Alkbh5*  *Fam177a* | Reverse: 5’ CTGACTGACCTTCTTGCTCTGCTG 3’  Forward: 5’ TGCAGCACCTCGGTCATTTA 3’  Reverse: 5’ TAACCCCACTTTCGCAAGCA 3’  Forward: 5’ GAAGGAGACACGACAGCAGTTGG 3’  Reverse: 5’ GCTTGTGACCTCTGCCTGATCTAC 3’  Forward: 5’ ATGAAGACGCTGTGCCACTGTG 3’  Reverse: 5’ CACGTTGTAGGCTGCTCTGCTC 3’  Forward: 5’ GCAAGGTGAAGAGCGGCATCC 3’  Reverse: 5’ GTCCACCGTGTGCTCGTTGTAC 3’  Forward: 5’ CAGCCTAGCACATGGTGACA 3’ | 87  115  104  128  241 |
|  | Reverse: 5’ AGGAAATCCACAGTGCTTCC 3’ |  |
| *Pcdhgb4* | Forward: 5’ TGAGTCAACCTCCCATCAGC 3’ | 147 |
|  | Reverse: 5’ ATGGCTTGCAGCATCTCTGT 3’ |  |
| *Tmem181c-ps* | Forward: 5’ ATACGGGTCCAGGGAGAGAG 3’ | 148 |
|  | Reverse: 5’ TTCCAGGGCTGACGTTTCAC 3’ |  |
| *Pcdhga 2* | Forward: 5’ CCCAACACTGACTGGCGT 3’ | 119 |
|  | Reverse: 5’ ATGGCTTGCAGCATCTCTGT 3’ |  |
| *Gpr176* | Forward: 5’ CGATGAGCAGACGTGAGGG 3’ | 424 |
|  | Reverse: 5’ ACAGACCACACTGGCACAAA 3’ |  |
| *Slc16a7* | Forward: 5’ TCTGACAACAGGTCACCTTC 3’ | 125 |
|  | Reverse: 5’ AAAGGACGCACAGACTACGA 3’ |  |
| *β-actin* | Forward: 5’ GTGCTATGTTGCTCTAGACTTCG 3’  Reverse: 5’ ATGCCACAGGATTCCATACC 3’ | 174 |
