# Supplementary material for: Downregulation of m6A Methyltransferase in the Hippocampus of Tyrobp–/– Mice and Implications for Learning and Memory Deficits
Source: Front Neurosci. 2022 Mar 21;16:739201. doi: 10.3389/fnins.2022.739201 (PMC8978996; doi:10.3389/fnins.2022.739201)
Supplement: Supplementary Table 2 — Information of quality control of the raw data. [file Table_2.DOCX]

**[Supplementary](https://www.ncbi.nlm.nih.gov/geo/query/acc.cgi?acc=GSE179827,%20supplementary) table 2. Information of quality control of the raw data.**

| Sample_ID | Raw  reads | Raw  bases | Valid reads | Valid  bases | Valid (%) | Q20  (%) | Q30 (%) | GC  (%) |
| --- | --- | --- | --- | --- | --- | --- | --- | --- |
| WT1 IP | 63258414 | 9.49G | 61189368 | 8.42G | 88.69 | 98.62 | 95.59 | 50.24 |
| WT2 IP | 70111114 | 10.52G | 66972696 | 9.34G | 88.84 | 97.15 | 92.31 | 49.16 |
| WT3 IP | 74177590 | 11.13G | 71579536 | 9.90G | 88.97 | 98.59 | 95.52 | 49.75 |
| *Tyrobp^-/-^*1 IP | 64581218 | 9.69G | 62814416 | 8.72G | 89.97 | 98.50 | 95.28 | 49.33 |
| *Tyrobp^-/-^*2 IP | 65432882 | 9.81G | 63295282 | 8.82G | 89.91 | 97.07 | 92.19 | 48.46 |
| *Tyrobp^-/-^*3 IP | 70342546 | 10.55G | 68297502 | 9.51G | 90.16 | 98.52 | 95.30 | 49.03 |
| WT1 input | 72104300 | 10.82G | 70350116 | 9.66G | 89.35 | 98.46 | 95.23 | 47.63 |
| WT2 input | 74009440 | 11.10G | 71350220 | 9.89G | 89.09 | 96.98 | 92.18 | 47.80 |
| WT3 input | 75560816 | 11.33G | 73554506 | 10.15G | 89.55 | 98.44 | 95.23 | 47.98 |
| *Tyrobp^-/-^*1 input | 69496210 | 10.42G | 67033990 | 9.25G | 88.75 | 98.27 | 94.96 | 48.00 |
| *Tyrobp^-/-^*2 input | 71328172 | 10.70G | 68879896 | 9.56G | 89.34 | 97.12 | 92.40 | 47.94 |
| *Tyrobp^-/-^*3 input | 71350674 | 10.70G | 69188154 | 9.56G | 89.35 | 98.39 | 95.14 | 47.59 |
